# A Living Database of HIV Implementation Research (LIVE Project): Protocol for Rapid Living Reviews

**DOI:** 10.2196/37070

**Published:** 2022-10-05

**Authors:** Ingrid Eshun-Wilson, Nathan Ford, Noelle Le Tourneau, Stefan Baral, Sheree Schwartz, Christopher Kemp, Elvin Geng

**Affiliations:** 1 Division of Infectious Diseases School of Medicine Washington University in St. Louis St. Louis, MO United States; 2 Department of Global HIV, Hepatitis and Sexually Transmitted Infections Programmes World Health Organization Geneva Switzerland; 3 Bloomberg School of Public Health Johns Hopkins University Baltimore, MD United States

**Keywords:** living review, HIV, systematic review, rapid review, implementation, HIV infection

## Abstract

**Background:**

HIV implementation research evolves rapidly and is often complex and poorly characterized, which makes the synthesis of data on HIV implementation strategies inherently difficult. This is further compromised by prolonged data abstraction processes due to variable interventions, outcomes, and context, and delays in the publication of review findings; this can all result in outdated and irrelevant systematic reviews.

**Objective:**

The LIVE project (A Living Database of HIV Implementation Research) aims to overcome these challenges by applying an implementation science lens to the conduct of rapid living systematic reviews and meta-analyses to inform HIV service delivery priorities and guideline development.

**Methods:**

The LIVE project will generate a series of living systematic reviews exploring implementation strategies for improving HIV cascade outcomes (HIV infection, HIV diagnosis, linkage and retention in HIV care, viral suppression, and mortality). We will search Embase and MEDLINE as well databases specific to review questions for studies conducted after 2004 using predefined search terms to identify studies conducted in any age group or setting, and using implementation strategies that target policy makers, society, health organizations, health workers, and beneficiaries of care and their families. Both randomized controlled trials and observational studies will be included to ensure reviews include pragmatic data. In addition to assessments of methodological quality, features of the implementation strategies, relevance for implementation, and evidence quality will be determined using recognized frameworks. After initial publication, knowledge gaps will be identified, and review questions and search strategies revised to address ongoing critical areas of inquiry. Updated searches will be conducted every 6 months, with subsequent ongoing screening, data abstraction, and revision of meta-analyses.

**Results:**

As of July 2022, five reviews are at various stages of development within the LIVE project. Three systematic reviews are underway and living review processes are in development for two reviews with estimated completion over the next 12 months.

**Conclusions:**

This project and resulting systematic reviews will provide critical insights for HIV service delivery to inform international guideline development.

**International Registered Report Identifier (IRRID):**

DERR1-10.2196/37070

## Introduction

Systematic reviews addressing HIV implementation research questions are challenged by difficulties in synthesizing heterogenous pragmatic research and can become outdated rapidly. As HIV prevention strategies, testing methods, and treatments become increasingly effective, current primary HIV research and evidence synthesis questions are refocusing on how best to implement effective interventions to ensure long-term sustained engagement in HIV care [1,2]. This continuous emergence of new implementation research means that traditional methods for generating “static” systematic reviews that may take months or years to produce can quickly become obsolete [3,4]. With each new guideline development cycle, new review teams, searches, protocols, and reviews are undertaken, resulting in substantial duplication of efforts, delays in the generation of synthesized evidence and inability for guideline developers to quickly update recommendations.

Living and rapid review methods have been developed in recent years (now catalyzed by the COVID-19 pandemic) to address these inefficiencies and increase the utility of review evidence; these methods have however been infrequently applied to HIV implementation research [5]. The field of HIV implementation science is a rapidly evolving field, with frequent changes to HIV service delivery approaches (eg, multi-month prescribing), drug delivery systems for HIV treatment (eg, long-acting antivirals), and HIV prevention (eg, vaginal rings) and testing modalities (eg, HIV self-testing). Living methods offer an approach for systematic review updating, where new evidence is incorporated into a review as it emerges, generating a continual updating process that maintains the relevance of synthesized findings and builds on previous work. Living reviews require an explicitly stated commitment to a predetermined frequency of searches and review updating [6]. Rapid reviews aim to accelerate the review process through the elimination or attenuation of some systematic review requirements, including searches in fewer databases, applying language or publication year restrictions, limiting gray literature searches, applying data mining processes, and altering duplicate screening, data extraction, and quality appraisal processes [7,8]. Rapid reviews are being conducted with increased frequency to respond to policy-making needs [9,10]. Rapid and living processes are ideal for incorporation into “living guidelines”—a dynamic guideline development process that, instead of conducting mechanistic guideline updates with a predetermined frequency, uses the results of continuous literature surveillance, rapid updating of prioritized reviews, and frequent virtual consultations with guideline panels to create a continuous guideline development and revision process; this helps to ensure that policy makers and health workers can make up-to-date, evidence-based public health decisions [11-14]. Accelerating the pace of evidence synthesis and dissemination can facilitate the early and effective adoption of new strategies for improving health and reduce the evidence-practice gap [15,16].

Heterogeneity, a frequent and desirable property of implementation research, further complicates evidence synthesis for HIV service delivery. The application of systematic review and meta-analytic methods—originally designed for homogenous efficacy data—to complex implementation research questions can result in systematic review findings that are of limited relevance to policy makers [17-19]. Establishing the effectiveness of strategies to increase HIV testing or antiretroviral therapy uptake and adherence requires detailed characterization of strategy features (eg, where, how, and who delivered the intervention) as well as incorporation of pragmatic data that establishes effectiveness under real-world conditions. Tools are available for characterization of implementation strategies, assessment of real-world relevance of primary research, and reporting of implementation research methods and results, but to date such tools have had limited application in HIV implementation research evidence synthesis [20-23]. Heterogeneity does not preclude evidence synthesis; it is important to develop approaches to accommodate varied study designs and implementation strategies and still draw conclusions from the evidence.

The Living Database of HIV Implementation Research (LIVE) project aims to generate a series of methodologically robust rapid and living reviews characterizing and evaluating the effects of HIV implementation strategies on HIV cascade outcomes through an ongoing process of data abstraction and frequent review updates to produce valid and relevant synthesized evidence that contributes to a rapid public health response to HIV. In addition, this work will identify evidence gaps and put forward new approaches for reviewing and meta-analyzing complex implementation research specific to HIV but with relevance to evidence synthesis in the implementation science field more broadly.

## Methods

This project protocol was designed according to PRISMA-P (Preferred Reporting Items for Systematic Review and Meta-Analysis Protocols) guidelines, living review guidelines, and World Health Organization (WHO) and Cochrane rapid review guidelines [6,8,10,24].

### Identification of Review Questions

Relevant HIV implementation science questions will be developed in consultation with HIV guideline development groups. This will include questions regarding effectiveness of HIV implementation strategies. Individual review protocols will be published on PROSPERO, the international prospective register of systematic reviews.

### Eligibility Criteria

Studies eligible for inclusion in living rapid reviews include those conducted in any population group or age category from any setting. Randomized controlled trials (RCTs), cohort studies (with or without a comparison arm), cross-sectional studies, and natural experiments are eligible for inclusion. Incorporation of a broad range of study designs including both randomized controlled trials and observational studies will facilitate exploration of the broad spectrum of implementation research assessing the performance of implementation strategies under trial and real-world conditions.

Studies must evaluate the implementation of evidence-based HIV interventions (strategies aimed at implementing a change to the way HIV testing, antiretroviral treatment [ART], or prevention is delivered to modify patient behavior and improve outcomes) and report on at least one HIV cascade outcome (HIV incidence, HIV testing uptake, ART initiation, ART adherence, viral suppression, retention in care) (Table 1). Eligible studies will be restricted to English language publications.

**Table 1:** Eligibility criteria for inclusion in LIVE rapid living reviews.

	Eligibility criteria
Population	All settings, all ages
Implementation strategy	Implementation strategy aimed at (1) implementing a change to the way HIV care and preventions strategies are delivered or (2) modifying patient behavior
Comparison	Other intervention, standard of care, or no comparison
Outcome	HIV incidence, HIV testing uptake, antiretroviral therapy initiation, antiretroviral therapy adherence, viral suppression, retention in care

### Database Searches

An information specialist will conduct searches of a minimum of two databases—MEDLINE and Embase—and will include CINAHL and other databases depending on the considered added value for the specified review question as determined in consultation with an information specialist. Search outputs will be refined through an iterative process of cross-checking against known studies in the field. Once finalized, automated searches running at a predetermined frequency (initially every 6 months) will generate updated lists of studies for screening and eligibility assessment and abstraction. Searches will include studies published between 2004 to the day of the search, but may be restricted to more recent studies depending on the specified review question.

### Gray Literature Searches

At minimum, conference abstracts of the International AIDS Society and the Conference on Retroviruses and Opportunistic Infections will be searched for the previous two years. Additional conference searches will depend on their relevance to review questions. Clinical trial registries including ClinicalTrials.gov and WHO International Clinical Trials Registry Platform registries will be searched routinely; depending on the specific review, further trial registries may be considered.

### Screening

Several team members may be involved in screening processes. Abstract and full-text screening will be conducted using Covidence software [25]. For abstract screening, 2 team members will screen the first 20% of abstracts with conflict resolution; once approaches to screening are calibrated and consistency developed, ongoing abstract screening will be conducted by one team member. Full-text screening will be conducted by one team member and excluded full texts will be screened by a second. Conference and clinical trial registry searches will be conducted by one team member with confirmation of eligibility of included abstracts by a second.

### Data Abstraction

Study data will be abstracted into the LIVE database hosted on the Airtable platform (a relational database designed to be easily modified by end users and widely used commercially [26]. Extracted study outcomes will include numerators and denominators as well as adjusted and unadjusted effect estimates. Data abstraction and methodological quality assessments will be conducted by one team member and reviewed by a second team member. Descriptive information will be extracted from each study (including details on publication, study design, setting, context, and demographic characteristics) and additional data regarding the critical characteristics and components of implementation strategies will be recorded using existing frameworks for evaluating characteristics of implementation strategies, reporting of implementation outcomes, assessments of real-world relevance of primary research, and implementation characteristics of trial design (Table 2). By applying these implementation science tools and frameworks, the LIVE project will employ evidence synthesis methods that accommodate complexity, recognizing that heterogeneity is an inherent feature of the current HIV response and is essential [1].

**Table 2:** Tools used to assess study quality and characterize intervention strategies for living rapid reviews.

Assessment tool	Purpose
Cochrane risk of bias tools [27,28]	Assess the methodological quality of randomized controlled trials
Newcastle Ottawa scale [29]	Assess the methodological quality of cohort and cross-sectional studies
Proctor implementation strategy framework [20]	Characterize implementation strategies
Proctor implementation outcome classification system [21]	Characterize and assess reporting implementation outcomes
Pragmatic explanatory continuum indicator summary (PRECIS)-2 tool [22]	Evaluate explanatory vs pragmatic approaches of studies
Curran effectiveness-implementation hybrid trial designs [30]	Characterize trial types based on focus: clinical effectiveness versus implementation

### Analyses

We will characterize individual study populations, implementation interventions, comparisons, and HIV cascade outcomes and other outcomes relevant to the review questions including harms and unintended consequences. We will use funnel plots to explore publication bias. If there is sufficient quantitative data, these data will be meta-analyzed in R, Stata, or SAS programs, depending on the type of data available for analysis (eg, continuous, binary, incidence, adjusted effect estimates, single means, or proportions). Pooled results and forest plots for random effects will be generated using Mantel-Hansel, Peto, generalized linear models, or generic inverse variance [31]. Inconsistency will be reviewed qualitatively to detect clinical diversity (population, context, implementation strategy) or methodological diversity (risk of bias, study design), and quantitatively using *I*^2^, Kendall τ statistics, and subgroup analysis. Decisions regarding the appropriateness of pooling data, subgrouping, and sensitivity analyses will be conducted by study teams and will follow guidelines as set out by the Cochrane Handbook. Given the inherently heterogenous nature of HIV implementation research, we anticipate substantial explained and unexplained heterogeneity; as a result, pooled estimates may in many cases not reflect one true population effect estimate relevant to all contexts but rather a broader assessment of overall benefit or harm across various contexts [32]. Where sufficient data are available, we will use meta-regression to explore heterogeneity.

In addition, where multiple strategies are presented, network meta-analyses (NMA) may be conducted and will follow guidelines for conduct and reporting of NMA. The frequentist or Bayesian NMA approaches will be used to generate networks, evaluate inconsistency, and rank interventions. Although the inherent nature of implementation strategies may in some cases violate the assumption of transitivity due to variability in context and strategy heterogeneity—in terms of design and fidelity to intervention delivery—this analytic technique allows for the comparison of multiple interventions that have not been compared directly due to public health urgency and resource constraints [33].

Where data are insufficient for meta-analysis, we will summarize data narratively. The overall confidence in the review findings will be evaluated using recognized methodologies for rating evidence certainty such as the Grading of Recommendations Assessment, Development, and Evaluation system [34].

### Living Processes

Once a review is completed and published, a continuous living process will be adopted to keep the review findings up to date as required [6,35]. First, the systematic review question will be examined in the light of the primary review findings and in consultation with key stakeholders (eg, WHO guideline developers) to determine if the question remains relevant in its current format, whether the review question should be altered to address different population groups, and whether additional strategies or specific implementation or HIV cascade outcomes should be focused on. Search strategies will be examined and refined to ensure that all relevant new terms and databases are included in updated search strategies. A comprehensive systematic search will be conducted every 6 months. If no new studies are detected, review records will be updated with the most recent search date and specify that no new relevant studies have been identified. If new studies are identified but appear unlikely to change the review findings or are insufficient for new meta-analyses, study data will be extracted but no meta-analyses will be performed. If new findings are deemed critical for revised or updated guidelines, new meta-analyses of all studies identified to date will be conducted and published in a peer reviewed journal. With each 6-month cycle, considerations for retirement of reviews will be revised, as the importance of research questions will be expected to change over time [36]. Such reviews may contribute to living guidelines, an emerging methodological area where guidelines are continuously assessed to determine whether they are sufficiently up to date and whether new studies or information is available that may change the guideline, leading to cycles of refinement and revision or retirement [14].

## Results

As of July 2022, five reviews are at various stages of development within the LIVE project. Data extraction is underway for 3 systematic reviews with the aim of completion by the end of December 2022; living review processes are under development for 2 reviews.

## Discussion

The LIVE project seeks to enhance the use of implementation research to inform guideline development and ultimately policy making. The project proposes to produce “living” systematic reviews by applying an ongoing updating and data extraction process to support guideline developers, including but not limited to questions on HIV service delivery at the WHO. In this project protocol, we outline a plan to support ongoing guideline development processes in HIV testing and use of antiretrovirals, but also identify how through the maintenance of living reviews this work can contribute to the future conceptualization and development of “living guideline” processes.

The additional application of implementation research tools and taxonomies further position this work to contribute to guidelines that directly impact global implementation efforts, particularly for questions in HIV service delivery. By broadly exploring how, where, and for whom HIV implementation strategies are most effective, the LIVE project will advance the implementation science field by directly addressing inherent heterogeneity and intervention complexity in implementation science evidence synthesis and support future HIV service delivery guideline development.

This work may be limited by difficulties in maintaining reviewers over the long term and ensuring continuous updates; the project will work to overcome this by involving a broad review team to ensure the ongoing longevity of individual systematic reviews. A further challenge may be decisions regarding when to publish an updated version of a review, retire a review, or alter review questions. To address this, decisions regarding review updates will be determined in close collaboration with experts and policy makers to ensure ongoing relevance.

Synthesizing implementation research evidence is complex. This protocol and review portfolio propose new directions for implementation science evidence synthesis that also have relevance for other implementation questions beyond HIV service delivery.

## Abbreviations

ART: antiretroviral therapy
LIVE: Living Database of HIV Implementation Research
NMA: network meta-analysis
PRISMA-P: Preferred Reporting Items for Systematic Review and Meta-Analysis Protocols
RCT: randomized controlled trial
WHO: World Health Organization
